# Flexible Sulphur

**Published:** 1869-02

**Authors:** 


Flexible Sulphur.—By adding to pure sulphur a four-hundredth part of chlorine or iodine it becomes very soft, so that it may be spread in thin leaves as flexible as leaves of wax.

